# Optically Activated 3D Thin‐Shell TiO_2_ for Super‐Sensitive Chemoresistive Responses: Toward Visible Light Activation

**DOI:** 10.1002/advs.202001883

**Published:** 2020-12-03

**Authors:** Donghwi Cho, Jun Min Suh, Sang‐Hyeon Nam, Seo Yun Park, Minsu Park, Tae Hyung Lee, Kyoung Soon Choi, Jinho Lee, Changui Ahn, Ho Won Jang, Young‐Seok Shim, Seokwoo Jeon

**Affiliations:** ^1^ Department of Materials Science and Engineering Center for Bio‐Integrated Electronics at the Simpson Querrey Institute for BioNanotechnology Northwestern University Evanston IL 60208 USA; ^2^ Department of Materials Science and Engineering Research Institute of Advanced Materials Seoul National University Seoul 08826 Republic of Korea; ^3^ Department of Materials Science and Engineering KAIST Institute for the Nanocentury Korea Advanced Institute of Science and Technology (KAIST) Daejeon 34141 Republic of Korea; ^4^ National research Facilities and Equipment Center (NFEC) Korea Basic Science Institute (KBSI) Daejeon 34133 Republic of Korea; ^5^ Engineering Ceramic Center Korea Institute of Ceramic Engineering and Technology Icheon 17303 Republic of Korea; ^6^ Division of Materials Science and Engineering Silla University Busan 46958 Republic of Korea

**Keywords:** gas sensors, light scattering, 3D nanostructures, room temperature, titanium dioxide

## Abstract

One of the well‐known strategies for achieving high‐performance light‐activated gas sensors is to design a nanostructure for effective surface responses with its geometric advances. However, no study has gone beyond the benefits of the large surface area and provided fundamental strategies to offer a rational structure for increasing their optical and chemical performances. Here, a new class of UV‐activated sensing nanoarchitecture made of highly periodic 3D TiO_2_, which facilitates 55 times enhanced light absorption by confining the incident light in the nanostructure, is prepared as an active gas channel. The key parameters, such as the total 3D TiO_2_ film and thin‐shell thicknesses, are precisely optimized by finite element analysis. Collectively, this fundamental design leads to ultrahigh chemoresistive response to NO_2_ with a theoretical detection limit of ≈200 ppt. The demonstration of high responses with visible light illumination proposes a future perspective for light‐activated gas sensors based on semiconducting oxides.

## Graphical abstract

The high‐performance light‐activated NO_2_ gas sensors using 3D TiO_2_ nanostructures are achieved by the unique light harvesting properties of the nanoporous structures. The light scattering effects from optical maze‐like nanostructures and numerous intermediate energy states from atomic layer deposition of the film electronically activate 3D TiO_2_ and enhance its gas sensing performance under UV illumination.

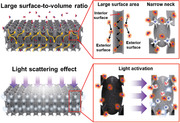

The semiconductor or chemoresistive‐type gas sensor based on metal oxides has been one of the most intensively studied and developed technologies with a growing demand from the internet of everything (IoE) market.^[^ ^1^ ^]^ The IoE application requires the gas sensors to exhibit a low power consumption for their installation in mobile devices, not to mention an excellent gas‐sensing performance. To achieve low power consumption, the heaters, more than anything else, should be removed from the current chemoresistive‐type gas sensor structures. The heaters operate at ≈200–400 °C with temperature uncertainty, thermally degrade the neighboring electronic components during operation, raise issues upon the miniaturization of device, and have also been a primary reason for the low reliability of the current sensor systems.^[^ ^2^ ^]^ Therefore, numerous studies have focused on discovering alternative materials capable of operation at near room‐temperature^[^ ^3^, ^4^ ^]^ or other chemical‐activation routes for sensing that consume less power than the thermal heaters.^[^ ^5^, ^6^ ^]^

One of the notable research directions in low temperature gas sensors is the use of light sources, particularly light‐emitting diodes (LEDs), for energy‐efficient activation of semiconducting sensor materials. Light sources with higher energy than the bandgap energy of the semiconductors can excite internal charge carriers to participate in the chemical reaction and accelerate the interaction between the semiconductor and the gas molecules. The previous studies on light‐activated gas sensors have repeatedly reported relatively improved gas responses and faster recovery characteristics under light illumination.^[^ ^7^, ^8^ ^]^ Considering the high‐performance requirements for gas sensors in IoE applications, further modifications of the sensory materials, other than just relying on their bulk properties, should precede the full utilization of the given light energy. In response, there have been numerous efforts reported to date with various strategies for light‐activated gas sensors including i) heterojunction engineering,^[^ ^9^ ^]^ ii) noble metal decoration,^[^ ^10^ ^]^ iii) utilization of nonoxide materials (2D, inorganic perovskites),^[^ ^11^, ^12^ ^]^ iv) incorporation of plasmonic nanoparticles,^[^ ^13^ ^]^ and v) development of effective nanostructures.^[^ ^14^ ^]^ Despite their active gas‐sensing performances, few studies suggest the possibility of improving the sensor's performance by adding structural factors to maximize the optoelectronic properties of the light‐activated gas sensors.

In this study, we systematically designed highly periodic 3D thin‐shell TiO_2_ nanostructures (denoted as “3D TiO_2_”) whose symmetry is body‐centered tetragonal (BCT) and the shell thickness is 30 nm. The nanostructure can make unique use of light by multiple scatterings within the optimal total thickness of 6 µm, which is first proven by finite element analysis (FEA) of the electromagnetic field (E‐field) distribution over 3D TiO_2_ by unit cell modeling. The optimized light‐scattering effects by 3D thin‐shell TiO_2_ on the enhancement of NO_2_ gas‐sensing performance were verified through the measurement of resistance change. The thin‐shell and film thickness of 3D TiO_2_ were precisely controlled for the optimization (thin‐shell thickness from 30 to 100 nm and film thickness from 3 to 15 µm, respectively). As a result, the fabricated 3D TiO_2_ exhibited significantly increased light absorption compared to that of the planar TiO_2_ thin film, with a thickness of 6 µm, yielding dramatically enhanced light‐activated gas responses with faster and complete recovery characteristics toward 5 ppm NO_2_. More interestingly, the gas response was further enhanced up to 4 times under a relative humidity of 50% due to the neutralization of hydroxyl groups under UV illumination.^[^ ^15^, ^16^ ^]^ Finally, a notable observation is that the fabricated 3D TiO_2_ exhibited light‐activated characteristics even under visible light illumination due to the defective nature of the atomic layer deposition (ALD)‐coated TiO_2_ thin‐shell, such as O vacancies, interstitial Ti, and carbon residues. To the best of our knowledge, there has been no systematic attempt to design a structural platform for the effective utilization of the illuminated LED light (<800 µW) for gas sensor applications operating in a highly humid environment at room temperature. The light‐activated, ultrasensitive (theoretical detection limit down to ≈202 parts per trillion (ppt) level) gas‐sensing properties of 3D TiO_2_ proven by the optical simulation in this study will certainly open a new perspective toward future chemoresistive‐type gas sensors operating at room temperature without any heating unit.

**Figure** **1**a presents the overall experimental approach used in this study and the developed highly periodic 3D TiO_2_. In brief, the optic system with a conformal phase mask for 3D nanofabrication, which generates light diffraction and interference in a proximity‐field, provides 3D periodic intensity distribution of the incident light through a prefabricated photopolymer thin film by the Talbot effect (Figure S1, Supporting Information).^[^ ^17^, ^18^, ^19^, ^20^ ^]^ The details can be found in the Experimental Section. Compared to other competing techniques such as self‐assembly, electrospinning, and many other bottom‐up methods, one of the notable technical advantages of this microelectromechanical system‐integrated method is the direct patterning of 3D scaffolds on the electrodes.^[^ ^21^, ^22^, ^23^ ^]^ Therefore, the further TiO_2_ deposition using ALD can be done without any additional transferring steps, which generally lead to poor contact issues between the electrodes and the deposited functional materials.^[^ ^24^, ^25^ ^]^ To apply 3D TiO_2_ to gas sensors, we fabricated 3D TiO_2_ on a SiO_2_/Si substrate with Pt interdigitated electrodes (IDEs). Figure 1b shows schematic illustrations and photographs of the 3D TiO_2_ on the IDEs‐patterned SiO_2_/Si substrates and gas‐sensing measurement system, respectively. The active area is 1 × 1 mm^2^ and the number of interdigitated fingers is 20 with 5 µm interspacing. The cross‐sectional scanning electron microscopy (SEM) images of the fabricated 3D TiO_2_ on a SiO_2_/Si substrate with Pt IDEs show the direct construction of the sensory material without any structural degradation, such as collapse and delamination (Figure 1c). For a clear observation of 3D TiO_2_, cross‐sectional high‐resolution transmission electron microscopy (HR‐TEM) images were carefully obtained, and they present a highly periodic BCT symmetry of 3D TiO_2_ (inset in Figure 1c). In addition, the in situ elemental mapping by energy‐dispersive X‐ray spectroscopy (EDS) proves the uniform deposition of TiO_2_ over the complicated 3D nanostructure during the ALD procedure, resulting in a monolithic 3D TiO_2_ nanostructure (Figure 1d,e). After the heating process for the polymeric template removal and the annealing of TiO_2_ in a single step,^[^ ^24^, ^25^ ^]^ 3D TiO_2_ resulted in the anatase phase. Likewise, the observed lattice fringes of 0.17 nm for d(105) and 0.27 nm for d(110) with a fast Fourier transform (FFT) pattern in Figure 1f indicate the anatase phase (JCPDS #21‐1272) and correspond to the X‐ray diffraction (XRD) analysis in Figure 1g.

**Figure 1:**
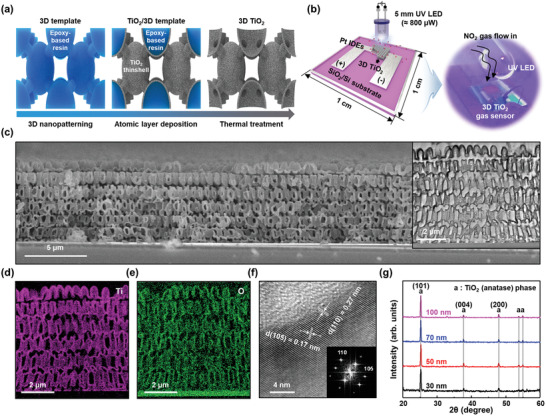
Design concept of and fabrication procedures for 3D TiO_2_ gas sensor. a) Schematic illustrations showing the fabrication of a 3D TiO_2_ gas sensor. b) Schematic illustrations and photographs of 3D TiO_2_ on the Pt IDEs patterned SiO_2_/Si substrates and gas‐sensing measurement system. c) Cross‐sectional SEM image of 3D TiO_2_ with 100 nm TiO_2_ thin‐shell thickness. The inset shows the HR‐TEM image of 3D TiO_2_. In situ elemental mapping results of d) Ti and e) O from the inset in (c) analyzed by EDS. f) FFT and lattice fringe images of the 3D TiO_2_. g) Comparison of XRD results of the fabricated 3D TiO_2_ with thin‐shell thicknesses of 30, 50, 70, and 100 nm, respectively.

The resultant 3D periodic porous TiO_2_ thin‐shell network with the periodicity of 600 nm offers the three major factors for ideal gas‐sensing performance: i) utility factor, ii) transducer function, and iii) receptor function.^[^ ^26^ ^]^ As the BCT unit cell of 3D TiO_2_ consists of two types of embodiments, a hollow ellipsoid core (center) and eight internecks connecting with the neighboring 1/8 parts of the hollow ellipsoids, these thin‐shell internecks allow the target gases to access both the exterior and interior surfaces of the 3D nanostructure (utility factor). It is noted that Sanger et al. reported the contribution of the hollow tubular structure with high‐aspect‐ratio to the significant improvement of the NO_2_ molecule collision frequency, which directly corresponds with our unprecedented enhancement of the sensing performances.^[^ ^27^ ^]^ In addition, the internecks in the unit cell also play an important role in the enhancement of the semiconducting performances of the TiO_2._ The junctions between the TiO_2_ thin‐shell networks narrow the conduction channels with double‐Schottky junctions originated from the depletion layers formed by the adsorption of ionized oxygen from the air.^[^ ^28^ ^]^ The internecks further narrow the conduction channel, which significantly enhances a transducer function that contributes to more efficient modulation of the electrical resistance upon exposure to target gases (**Figure** **2**a). For the receptor function, the light‐scattering effects induced by the 3D nanostructure facilitate the significantly improved photoactivation of 3D TiO_2_, resulting in numerous photogenerated electron–hole pairs for reaction with NO_2_ molecules (Figure 2b).

**Figure 2:**
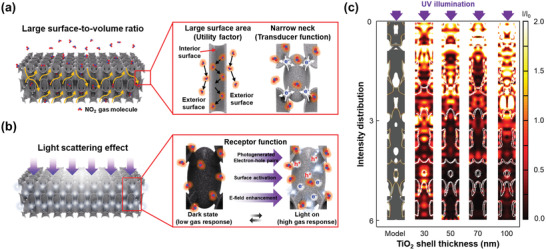
The geometric advances of 3D TiO_2_ gas sensor for detecting NO_2_ gas molecules. Schematic illustrations of a) highly periodic TiO_2_ nanonetworks for effective NO_2_ gas permeation and b) light‐scattering effects induced from 3D nanostructures enhancing E‐field of the incident UV light. c) Comparison of the E‐field intensity distributions when UV light passes through 3D TiO_2_ simulated by finite element modeling as a function of TiO_2_ thin‐shell thickness.

To effectively utilize the large surface area of 3D TiO_2_ in terms of the light‐activated gas sensors, the total film thickness of 3D TiO_2_ and the TiO_2_ thin‐shell thickness should be carefully defined. In other words, it is essential to find the optimal thickness that can take full advantage of UV activation for high gas sensing performance. As the film thickness of 3D TiO_2_ increases, the numerous repeatedly existing layers in the tri‐axial directions of 3D TiO_2_ gradually inhibit both an effective photoactivation and gaseous passage. The thicker TiO_2_ thin‐shell reduces the interspace between TiO_2_ thin‐shells and the porosity of the overall film, while its light absorption characteristics are expected to be improved due to the decreased transmittance through the TiO_2_ nanostructure. To find an optimal combination of the above conditions, the film thickness of 3D TiO_2_ was controlled as 3, 6, and 15 µm, and the TiO_2_ thin‐shell thickness was controlled as 30, 50, 70, and 100 nm. The dependencies of both thicknesses of 3D TiO_2_ in terms of light‐scattering effects were investigated by defining the unit cell models for each case using a commercial finite‐difference time‐domain (FDTD) simulation software (Lumerical). It is noteworthy that this numerical approach for finding the optimal conditions was valid owing to the exceptional structural uniformity, which is one of the distinctions of this work. Overall simulation data dependent on the film thickness of the 3D TiO_2_ (Figure S2a, Supporting Information) confirms that the E‐field enhancement in UV by the 3D TiO_2_ is significantly improved owing to its optical maze‐like architecture that leads to effective UV light absorption. In particular, the periodicity of the structure and the refractive index mismatch at the interfaces between air (refractive index ≈ 1) and TiO_2_ (refractive index ≈ 1.7) induce the light interference resulting in an active E‐field enhancement along the 3D nanostructure. In fact, there is a saturation point to utilize the incident UV light through 3D TiO_2_ because of the considerable scattering effect from a thicker 3D nanostructure. For example, the degree of E‐field enhancement in UV intensively increases until the total thickness reached 6 µm, but beyond that total thickness, this effect is saturated at ≈70% (Figure S2b, Supporting Information). It indicates that the excess of film thickness over 6 µm is not useful for photoactivation. Thus, there should be an effective total thickness between 3 and 15 µm, and it is controlled as 6 µm in this work.

The TiO_2_ thin‐shell thickness affects the effective refractive index^[^ ^29^ ^]^ and porosity of 3D TiO_2_,^[^ ^24^ ^]^ which significantly contribute to both the E‐field enhancement and gas accessibility. Thus, we controlled the TiO_2_ thin‐shell thickness in the range from 30 to 100 nm by varying the deposition cycles of the ALD. The variation of light‐matter interaction from the controlled TiO_2_ thin‐shell thicknesses generates complex E‐field intensity distributions (Figure 2c). As the thin‐shell thicknesses become thicker, the deeper regions of 3D TiO_2_ have relatively weaker intensities due to decreased transmission of light. For a clear understanding of the E‐field enhancement effect on UV absorption, the total sum of intensity for 3D TiO_2_ (film thickness 6 µm as the function of thin‐shell thickness) and planar TiO_2_ thin films are calculated with each thin‐shell thickness (Figure S3, Supporting Information). The light confinement is defined as the sum of intensity values in 5 nm cubic voxels consisting of TiO_2_ regions in the FDTD simulation. As the thin‐shell thickness increases, the increments in total E‐field are more prominent for 3D TiO_2_ than for the planar TiO_2_ films, which is originated from the porosity differences between the planar TiO_2_ thin films and 3D TiO_2_. Therefore, the overall light‐activated gas‐sensing performance should be determined considering both the nanostructural and light‐scattering effects.

After the fabrication of 3D TiO_2_ using various combinations with the total film thickness of 3, 6, and 15 µm and thin‐shell thickness of 30, 50, 70, and 100 nm, 3D TiO_2_ was exposed to 5 ppm NO_2_ under ultraviolet (UV) illumination at room temperature to investigate its gas‐sensing properties. An illumination power to the sample of less than 800 µW from a commercial 5 mm UV micro‐LED was sufficient to photoactivate the sensor. It is worth mentioning that no illumination result on TiO_2_ chemoresistors has ever achieved high performance under this illumination condition. The extremely low energy consumption is a key factor for miniaturization and integration to IoE devices. **Figure** **3**a,b shows top‐view and cross‐sectional view of SEM images of 6 µm thick 3D TiO_2_ with 30, 50, 70, and 100 nm thin‐shell thickness. A clear decrease in porosity can be verified for the thicker TiO_2_ thin‐shells and is expected to significantly affect the gas‐sensing properties along with the light‐scattering effects. Figure 3c–e shows the gas‐response curves to 5 ppm NO_2_ for each 3D TiO_2_. The resistance increased instantly upon the initial exposure to the NO_2_, which indicates the behavior of an n‐type semiconductor. Compared to the planar TiO_2_ thin film exhibiting almost no gas response under the dark condition and a very little response under UV illumination, 3D TiO_2_ exhibited significantly improved gas response and full recovery to the baseline resistance (Figure S4, Supporting Information). In addition, both samples exhibited decreased base resistance under UV illumination compared to that under dark condition, which indicates the photogeneration of electron–hole pairs. The trends in base resistance exhibited increment for thicker TiO_2_ thin‐shell thickness of each 3D TiO_2_ film up to 70 nm and decrement after 70 nm, except for 3D TiO_2_ with 15 µm, which exhibited continuous increment after 70 nm. This can be attributed to the effects of a noneffective dead bottom volume of 3D TiO_2_ with 15 µm that is previously mentioned (Figure S2, Supporting Information). As simulated in Figure 2; and Figure S3 (Supporting Information), 100 nm TiO_2_ thin‐shell thickness exhibited the highest field enhancement intensity for 3D TiO_2_ with 6 µm and this is expected to have contributed to resistance decrease along with nanostructural effects (porosity difference). Although the simulation on 3D TiO_2_ with 15 µm has not been conducted for all TiO_2_ thin‐shell thicknesses, their impressive field enhancement will certainly be limited to the upper region of 3D TiO_2_ with 15 µm and cannot contribute to the conduction channel at the bottom region. As a result, bouncing back to resistance decrement from resistance increment trends up to 70 nm TiO_2_ thin‐shell thickness, which is highly related to light scattering effects could not happen for 15 µm 3D TiO_2_. These trends have been reproducible and confirmed through the fabrication of multiple batches of 3D TiO_2_ samples. The gas response can be calculated dividing the changes in saturated resistance before and after exposure to NO_2_ (*R*_air_ and *R*_gas_, respectively) by the baseline resistance (*R*_air_), expressed as (*R*_gas_ − *R*_air_) / *R*_air_ × 100 (%).^[^ ^30^, ^31^ ^]^ The gas responses of 3D TiO_2_ with the different thickness combinations toward 5 ppm NO_2_ under UV illumination are summarized in Figure 3f. The total thickness of 6 µm exhibited higher gas response for all TiO_2_ thin‐shell thickness conditions than that for 3D TiO_2_ with 3 and 15 µm, which is well‐matched with the simulation data. For example, low response with nonstability was observed for the 15 µm sample because of the considerable volume of the unexposed region at the bottom, which is regarded as a dead volume. The gas responses kept decreasing as the TiO_2_ thin‐shell thickness increased up to 70 nm but increased again as it went up to 100 nm. As the TiO_2_ thin‐shell thickness increases, the volume fraction of vacancy compared to the TiO_2_ area, calculated from the top‐view SEM images in Figure 3a, decreases as shown in Figure 3g, indicating less porous nanostructures and less TiO_2_‐gas molecule interactions. Up to 70 nm, this nanostructural effect, a term defined as the changes in the utility factor (gas molecule accessibility) and transducer function (effective modulation of electric resistance) dominates the gas‐sensing properties of 3D TiO_2_, but for a thicker TiO_2_ thin‐shell, over 70 nm, it starts to become dominated by the light‐scattering effects from the complex 3D nanostructures. Thus, it is evident that both the levels of photoactivation and gas dynamics are crucial factors for the light‐activated gas sensors. Although the calculated total sum of intensity was the highest for 3D TiO_2_ with 100 nm thickness of thin‐shell, the overall gas‐sensing performance considering both the nanostructural effect and E‐field enhancement was the best for the 6 µm 3D TiO_2_ with 30 nm thin‐shell thickness. It has a nearly 55 times higher sum of intensity compared to that of the planar TiO_2_ thin film upon UV illumination, which is sufficient to take advantage of the complex 3D nanostructure designed in this study. In order to further study the strength of our 3D TiO_2_ nanostructures for the light activation, three other samples were prepared for the comparison, including TiO_2_ inverse opal (small and large overall thickness) and self‐assembled TiO_2_ nanoparticles. It is noted that the simulations assumed a highly ordered nanostructure. Compared with 3D TiO_2_, it is clearly observed that the E‐field enhancement occurs at the top region of the control structures due to their restricted light penetration depth from active reflection at the top of the structures. On the other hand, the incident light through 3D TiO_2_ reaches to the bottom of the structure and contributes to the utilization of the whole structure. This active light scattering through the entire 3D TiO_2_ leads to enhanced NO_2_ detection performances (Figures S5 and S6, Supporting Information). The highest response to 5 ppm NO_2_ under UV illumination was exhibited by TiO_2_ inverse opal with 1 µm thickness (385.88%) and it was just 2.12 times enhancement compared to dark condition. Considering our 3D TiO_2_ exhibiting a gas response of 3058.7% with 23.55 times enhancement by UV illumination, there is no significant light enhancement effects for the prepared three control samples.

**Figure 3:**
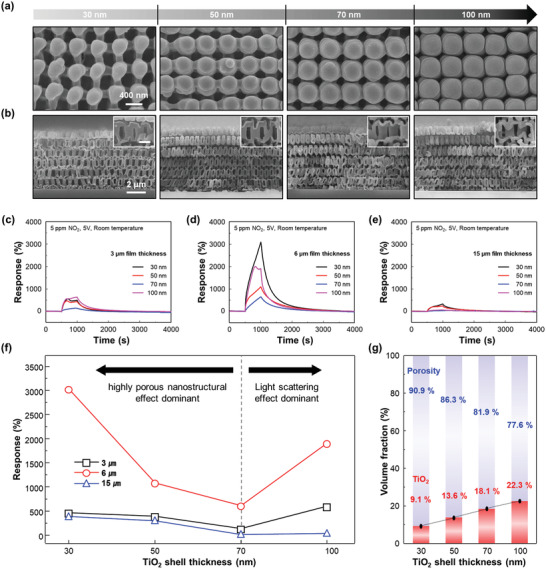
Optimization of total film thickness and TiO_2_ thin‐shell thickness. a) Top‐view and b) cross‐sectional SEM images of 3D TiO_2_ with increasing TiO_2_ thin‐shell thickness: 30, 50, 70, and 100 nm. Response transients of c) 3 µm, d) 6 µm, and e) 15 µm film thickness of the 3D TiO_2_ to 5 ppm NO_2_ under UV illumination as a function of controlled TiO_2_ thin‐shell thickness. f) Calculated responses of 3D TiO_2_ to 5 ppm NO_2_ under UV illumination with various TiO_2_ thin‐shell thickness and film thickness. g) Comparison plot for the volume fraction of air and TiO_2_, as a function of TiO_2_ thin‐shell thickness.

To verify further the gas‐sensing properties, such as stability and detection limit of 3D TiO_2_ with a total thickness of 6 µm and a thin‐shell thickness of 30 nm, the sample was exposed to multiple pulses of NO_2_ at various concentrations under UV illumination (**Figure** **4**a). The gas responses showed an excellent linear relationship (slope = 2.75 ppm^−1^) with gas concentration as described in Figure 4b. Although the lowest concentration of NO_2_ tested in this work was 400 ppb, the theoretical detection limit can be calculated by extrapolating the linear relationship to where the signal to noise ratio is equal to 3.^[^ ^32^ ^]^ Accordingly, the theoretical detection limit was calculated to be as low as 202 ppt, which may be advantageous for detection of extremely low concentration of target gases, such as those of explosives and narcotics. In Figure 4c, the measurement under a relative humidity of 50% (RH50) revealed the exceptional gas response of 3D TiO_2_ to 5 ppm NO_2_, as high as 12 200%. While exhibiting promising gas responses under UV illumination both at dry and humid conditions, a relatively slow response time (the time required to reach 90% of the saturated resistance under gas exposure, 428 s) might need to be improved for certain applications. In that case, some amount of external heating can accelerate the kinetics of gas molecules to yield faster response time, but this study will focus on light activation without any external heating.

**Figure 4:**
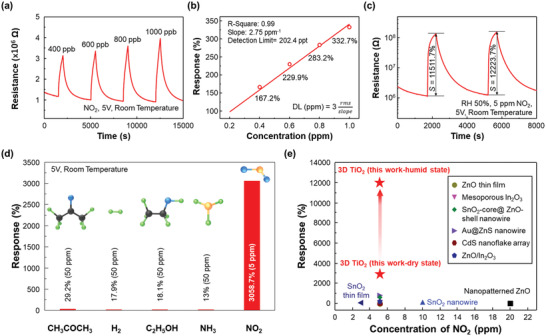
Sensing performances of 3D TiO_2_ gas sensors with 30 nm thin‐shell thickness and 6 µm film thickness. a) Response curves to different NO_2_ concentrations at 5.0 V. b) Linear fit of the responses as a function of NO_2_ concentration at 5.0 V. c) Resistance curve of the fabricated gas sensors under exposure of humidity of 50%. d) Selective NO_2_‐sensing performance of 3D TiO_2_ gas sensors. e) Comparison of gas‐sensing properties with those in other previous works.

The selectivity toward five different gas species (50 ppm CH_3_COCH_3_, H_2_, C_2_H_5_OH, NH_3_, and 5 ppm NO_2_) was tested on the 3D TiO_2_ sensor. As shown in Figure 4d, exceptionally selective detection of NO_2_, even with the lowest concentration, was achieved. It is well‐known that the absorbed ionized oxygen species (O_2_^−^, O^−^, O^2−^) on the surface of TiO_2_ are the key to the electronic interaction between 3D TiO_2_ and the gas molecules. Although oxygen molecules (O_2(gas)_) have very low chance to be ionized on the surface of TiO_2_ at room temperature,^[^ ^28^ ^]^ several studies have experimentally proved the existence of ionized oxygen species (O_2(ads)_^−^) on the surface of metal oxides even at room temperature by comparing conductance under either pure O_2_ and pure N_2_ without any photoactivation.^[^ ^7^, ^15^ ^]^ Under UV illumination, the O_2(ads)_^−^ recombine with photogenerated holes (h_(photo)_^+^) and become desorbed from the surface (h_(photo)_^+^ + O_2(ads)_^−^ ↔ O_2(gas)_). The desorption of O_2(gas)_ is considered advantageous to gas‐sensing properties as it supplies the available reactive sites of 3D TiO_2_, and the trapped electrons result in the decrease in resistance.^[^ ^7^, ^33^ ^]^ Meanwhile, some of the photogenerated electrons (e_(photo)_^−^) can interact with the adsorbed O_2(gas)_ to form photogenerated oxygen ions (O_2(gas)_ + e_(photo)_^−^ ↔ O_2(photo)_^−^). It should be clear that O_2(ads)_^−^ desorption would overwhelm O_2(photo)_^−^ adsorption. Otherwise, the resistance would not decrease under UV illumination.^[^ ^15^ ^]^ The O_2(photo)_^−^ is expected to be weakly bound to 3D TiO_2_ and have higher reactivity compared to the naturally chemisorbed O_2(ads)_^−^, which is thermally stable and less reactive at room temperature.^[^ ^34^ ^]^ Therefore, NO_2_ sensing and recovery are more likely to be accelerated under UV illumination (NO_2_ + O_2(photo)_^−^ ↔ NO_2(ads)_^−^ + O_2(gas)_) as demonstrated in the measurement. As there are already excessive photogenerated electrons trapped in TiO_2_, reducing gases like CH_3_COCH_3_, H_2_, C_2_H_5_OH, and NH_3_, which produce electrons when interacting with O_2(photo)_^−^, are less likely to exhibit as much improved gas response as an oxidizing gas such as NO_2_, which consumes electrons, resulting in high NO_2_ selectivity. When in humid condition, H_2_O molecules become ionized on the surface of TiO_2_ (H_2_O_(gas)_ + Ti + O_lattice_ ↔ (Ti^+^ – OH^−^) + (OH)_lattice_^+^ + e^−^).^[^ ^35^ ^]^ Following exposure to NO_2_, NO_2_ is adsorbed on the surface of 3D TiO_2_ ((Ti^+^ – OH^−^) + (OH)_lattice_^+^ + e^−^ + NO_2_ ↔ (Ti^+^ – NO_2_^−^) + H_2_O + O_lattice_).^[^ ^16^ ^]^ Under UV illumination, the neutralization of the hydroxyl groups (OH^−^) is possible with photogenerated holes (OH^−^ + h_(photo)_^+^ ↔ OH_(photo)_), and OH_(photo)_ can accelerate the above reaction and further improve NO_2_ gas response.^[^ ^15^ ^]^ That is the most‐widely accepted conduction mechanism under a moderate amount of humidity^[^ ^16^, ^36^, ^37^ ^]^ other than the hopping charge transport mechanism. When compared to other previously reported light‐activated gas sensors, the exceptional sensing performances of the fabricated 3D TiO_2_ greatly exceed them, as shown in Figure 4e.^[^ ^38^, ^39^, ^40^, ^41^, ^42^, ^43^, ^44^, ^45^, ^46^ ^]^ In terms of IoE application, the humidity‐boosted gas‐sensing capabilities can significantly contribute to the development of disease diagnosis devices, which collect disease biomarkers from humid sources, such as the exhaled breath.

Interestingly, 3D TiO_2_ exhibited unconventional absorbance at wavelengths longer than that corresponding to the intrinsic bandgap of TiO_2_, 3.2 eV, while the planar TiO_2_ thin film did not exhibit any absorbance at wavelengths longer than 380 nm (**Figure** **5**a). It is noted that the absorbance of all TiO_2_ samples were calculated by using the measured total transmittance for presenting enhanced light absorption due to 3D geometric advances. As the thin‐shell thickness of 3D TiO_2_ increases up to 100 nm, the unconventional light absorption of TiO_2_ in the visible light is enhanced (>626.2% for the 100 nm thickness at a wavelength of 500 nm). The absorbance of 3D TiO_2_ with normal transmittance, which contains both light scattering and actual light absorption effect, shows significant increase in broadband of visible light due to its considerable light scattering behaviors as an effective optical maze (Figure S7, Supporting Information). Meanwhile, when exposed to 5 ppm NO_2_ under visible light, 3D TiO_2_ exhibited improved gas‐sensing properties compared to that under dark conditions, which should not happen considering the bandgap energy of TiO_2_ (Figure 5b). The gas responses to red light (680 nm), green light (532 nm), and blue light (470 nm) were 295.9%, 103.5%, and 370.8%, respectively (Figure 5c), due to its structure‐induced light‐scattering effect, which was not proportional to the wavelength of the incident light.^[^ ^47^ ^]^ In other words, the visible light was actually absorbed by 3D TiO_2_ for the photoactivation.

**Figure 5:**
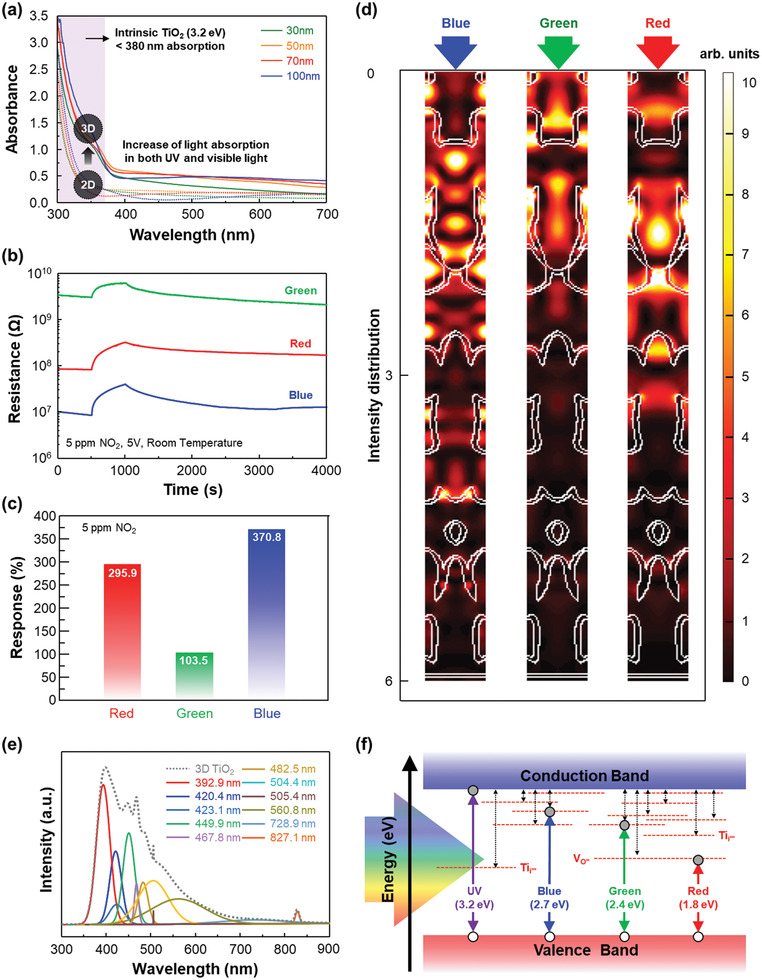
Unconventional NO_2_ gas response of 3D TiO_2_ under the illumination of visible light. a) UV–vis absorbance spectra of 3D TiO_2_ and planar TiO_2_ thin films with different thin‐shell thicknesses. The absorbance was calculated by using total transmittance. b) Resistance change curves and c) gas responses of the 3D TiO_2_ to 5 ppm of NO_2_ under LED irradiation of visible lights (red, green, and blue). d) Calculated E‐field intensity distributions through 3D TiO_2_ under illumination of various visible lights, such as blue (470 nm), green (532 nm), and red (680 nm), respectively. e) Photoluminescence spectra with λ_EX=295 nm for 3D TiO_2_. f) Reconstructed band diagram of 3D TiO_2_ from the measured PL data in (e).

To interpret these unusual photoactivation behaviors of 3D TiO_2_, further FDTD simulation was carried out to calculate the E‐field enhancement for each wavelength and transmission depending on the diffracted orders (Figure S8, Supporting Information). As shown in Figure 5d, the E‐field enhancement under blue light was the highest, that under red light was the second‐highest, and that under green light was the lowest, which corresponds to the sequence in gas response data. In other words, the defective nature of the ALD‐deposited 3D TiO_2_ provided an intermediate energy state in the forbidden zone of the 3D TiO_2_ band structure, and the excellent light scattering of 3D TiO_2_ leading to E‐field enhancement provided a high possibility of light absorption through the intermediate energy state. This would not have happened in a planar TiO_2_ thin film, which does not have any E‐field enhancement. To verify the intermediate energy state of 3D TiO_2_, photoluminescence (PL) spectra were acquired for the optimized 3D TiO_2_ (with total thickness of 6 µm and thin‐shell thickness of 30 nm) and unconventional photoluminescence at several wavelengths was identified (Figure S9, Supporting Information), indicating actual absorption of visible light wavelengths. Among the many deconvoluted peaks, the predominant emission peak at below 400 nm indicates the slightly shifted main peak of the TiO_2_ anatase phase, and the many extra peaks explain the intermediate energy states that correspond to various visible wavelengths (Figure 5e). According to the calculated intermediate energy states, the band diagram of 3D TiO_2_ was reconstructed as shown in Figure 5f. It can be explained by considering the interstitial Ti defects (Ti*_i_*) and oxygen vacancies (V_oS_) during the ALD procedure.^[^ ^48^ ^]^ According to the O 1*s* scan of the X‐ray photoelectron spectroscopy analysis, there are three major deconvoluted peaks originating from the lattice (530 eV), —OH (531 eV), and C=O (532 eV), respectively (Figure S10, Supporting Information). Comparing with the planar TiO_2_ thin film, the suppression of the C=O peak for 3D TiO_2_ proves the increase in the O vacancies. In addition, the inevitable existence of the carbon atom during the heat treatment provides the additional intermediate band states that ensure that the unique activation range is extended from UV to the wide band of the visible light range. Thus, the inherent defects of 3D TiO_2_ facilitated light‐activated gas‐sensing properties, even at the visible wavelength, with the help of the superb light‐scattering effects of the intricate 3D TiO_2_ nanostructures. This implies that the photoactivation under the desired wavelength could also be achieved without any attributes from defects, but through the systematic design of the band structure through various strategies, such as catalyst decoration or formation of heterojunctions.

In conclusion, the realization of the highly periodic 3D TiO_2_ is of fundamental significance to state‐of‐the‐art light‐activated gas sensor applications. The high degree of freedom to optimize the governing structural factors of the chemoresistive materials offers new design opportunities to overcome the bottlenecks of the conventional method for light‐activated gas sensors, which hinder the simultaneous employment of the gas dynamics and light utilization. Owing to its perfect structural uniformity, the two crucial factors, TiO_2_ thin‐shell thickness and total thickness, were precisely optimized as 30 nm and 6 µm, respectively, based on the FEA analysis. With these attributes, the open porous, interconnected 3D nanonetwork leads to significantly enhanced utilization of the incident light, which was experimentally verified with the exceptional NO_2_‐sensing performance having a theoretical detection limit of ≈200 ppt and stable operation at room temperature using low power consumption of a micro‐LED, less than 800 microwatts, for irradiation of not only UV but also visible lights. A remaining technical barrier in the application of this technology is the fabrication of denser 3D structures in terms of optical and chemical activities. The explorations of other symmetries, such as the wood‐pile structure, and material designs for controlling the band structure represent other promising research directions for the future. Therefore, the concept of the micro‐electromechanical system (MEMS) integrable 3D nanopatterning that can generate the effective light‐activated gas sensor platform presented in this work holds great promise as a rational strategy for developing general oxide‐based gas sensors in IoE applications.

## Conflict of Interest

The authors declare no conflict of interest.

## Supporting information

Supporting Information

## References

[1] R. A. Potyrailo , Chem. Rev. 2016, 116, 11877. 27602947 10.1021/acs.chemrev.6b00187

[2] R. Prajesh , N. Jain , A. Agarwal , Microsyst. Technol. 2016, 22, 2185.

[3] Y.‐S. Shim , K. C. Kwon , J. M. Suh , K. S. Choi , Y. G. Song , W. Sohn , S. Choi , K. Hong , J.‐M. Jeon , S.‐P. Hong , S. Kim , S. Y. Kim , C.‐Y. Kang , H. W. Jang , ACS Appl. Mater. Interfaces 2018, 10, 31594. 30136839 10.1021/acsami.8b08114

[4] K. C. Kwon , J. M. Suh , T. H. Lee , K. S. Choi , K. Hong , Y. G. Song , Y.‐S. Shim , M. Shokouhimehr , C.‐Y. Kang , S. Y. Kim , H. W. Jang , ACS Sens. 2019, 4, 678. 30799610 10.1021/acssensors.8b01526

[5] Y. H. Kim , S. J. Kim , Y.‐J. Kim , Y.‐S. Shim , S. Y. Kim , B. H. Hong , H. W. Jang , ACS Nano 2015, 9, 10453. 26321290 10.1021/acsnano.5b04680

[6] H. Chen , Y. Chen , H. Zhang , D. W. Zhang , P. Zhou , J. Huang , Adv. Funct. Mater. 2018, 28, 1801035.

[7] B. Liu , Y. Luo , K. Li , H. Wang , L. Gao , G. Duan , Adv. Mater. Interface 2019, 6, 1900376.

[8] M. M. Hashemi , A. Nikfarjam , H. Hajghassem , N. Salehifar , J. Phys. Chem. C 2020, 124, 322.

[9] H. Wang , L. Zhoi , Y. Liu , F. Liu , X. Liang , F. Liu , Y. Gao , X. Yan , G. Lu , Sens. Actuators, B 2020, 305, 127498.

[10] Q. A. Drmosh , A. H. Hendi , M. K. Hossain , Z. H. Yamani , R. A. Moqbel , A. Hezam , M. A. Gondal , Sens. Actuators, B 2019, 290, 666.

[11] H. Chen , M. Zhang , R. Bo , C. Barugkin , J. Zheng , Q. Ma , S. Huang , A. W. Y. Ho‐Baillie , K. R. Catchpole , A. Tricoli , Small 2018, 14, 1702571. 10.1002/smll.201702571 29280263

[12] D. Gu , X. Wang , W. Liu , X. Li , S. Lin , J. Wang , M. N. Rumyantseva , A. M. Gaskov , S. A. Akbar , Sens. Actuators, B 2020, 305, 127455.

[13] Y. Zhou , C. Zou , X. Lin , Y. Guo , Appl. Phys. Lett. 2018, 113, 082103.

[14] J. Cui , L. Shi , T. Xie , D. Wang , Y. Lin , Sens. Actuators, B 2016, 227, 220.

[15] L. Liu , X. Li , P. K. Dutta , J. Wang , Sens. Actuators, B 2013, 185, 1.

[16] Y. G. Song , Y.‐S. Shim , J. M. Suh , M.‐S. Noh , G. S. Kim , K. S. Choi , B. Jeong , S. Kim , H. W. Jang , B.‐K. Ju , C.‐Y. Kang , Small 2019, 15, 1902065. 10.1002/smll.201902065 31379070

[17] D. Cho , J. Park , J. Kim , T. Kim , J. Kim , I. Park , S. Jeon , ACS Appl. Mater. Interfaces 2017, 9, 17369. 28452466 10.1021/acsami.7b03052

[18] D. Cho , Y.‐S. Shim , J.‐W. Jung , S. Nam , S. Min , S.‐E. Lee , Y. Ham , K. Lee , J. Park , J. Shin , J.‐W. Hong , S. Jeon , Adv. Sci. 2020, 7, 1903708. 10.1002/advs.201903708 PMC7284194 32537413

[19] J. Park , K.‐I. Kim , K. Kim , D.‐C. Kim , D. Cho , J. H. Lee , S. Jeon , Adv. Mater. 2015, 27, 8000. 26524086 10.1002/adma.201503746

[20] S. Jeon , J.‐U. Park , R. Cirelli , S. Yang , C. E. Heitzman , P. V. Braun , P. J. A. Kenis , J. A. Rogers , Proc. Natl. Acad. Sci. USA 2004, 101, 12428. 15314211 10.1073/pnas.0403048101 PMC515078

[21] J. Zhang , Y. Li , X. Zhang , B. Yang , Adv. Mater. 2010, 22, 4249. 20803529 10.1002/adma.201000755

[22] M.‐H. Seo , J.‐Y. Yoo , M.‐S. Jo , J.‐B. Yoon , Adv. Mater. 2020, 32, 1907082. 10.1002/adma.201907082 32253800

[23] S.‐J. Choi , I.‐D. Kim , Electron. Mater. Lett. 2018, 14, 221.

[24] C. Ahn , J. Park , D. Kim , S. Jeon , Nanoscale 2013, 5, 10384. 24057038 10.1039/c3nr03115b

[25] S. Cho , C. Ahn , J. Park , S. Jeon , Nanoscale 2018, 10, 9747. 29767206 10.1039/c8nr02330a

[26] N. Yamazoe , G. Sakai , K. Shimanoe , Catal. Surv. Asia 2003, 7, 63.

[27] A. Sanger , S. B. Kang , M. H. Jeong , M. J. Im , I. Y. Choi , C. U. Kim , H. Lee , Y. M. Kwon , J. M. Baik , H. W. Jang , K. J. Choi , Adv. Sci. 2018, 5, 1800816. 10.1002/advs.201800816 PMC6145242 30250810

[28] N. Barsan , U. Weimar , J. Electroceram. 2001, 7, 143.

[29] X. A. Zhang , A. Bagal , E. C. Dandley , J. Zhao , C. J. Oldham , B.‐I. Wu , G. N. Parsons , C.‐H. Chang , Adv. Funct. Mater. 2015, 25, 6644.

[30] J.‐H. Lee , Sens. Actuators, B 2009, 140, 319.

[31] J. M. Suh , Y.‐S. Shim , D. H. Kim , W. Sohn , Y. Jung , S. Y. Lee , S. Choi , Y. H. Kim , J.‐M. Jeon , K. Hong , K. C. Kwon , S. Y. Park , C. Kim , J.‐H. Lee , C.‐Y. Kang , H. W. Jang , Adv. Mater. Technol. 2017, 2, 1600259.

[32] J. Li , Y. Lu , Q. Ye , M. Cinke , J. Han , M. Meyyappan , Nano Lett. 2003, 3, 929.

[33] R. Kumar , N. Goel , M. Kumar , ACS Sens. 2017, 2, 1744. 29090571 10.1021/acssensors.7b00731

[34] X. Tian , X. Yang , F. Yang , T. Qi , Colloid Surf. A 2019, 578, 123621.

[35] N. D. Chinh , C. Kim , D. Kim , J. Alloys Compd. 2019, 778, 247.

[36] P. Srinivasan , D. Prakalya , B. G. Jeyaprakash , J. Alloys Compd. 2020, 819, 152985.

[37] Q. Geng , X. Lin , R. Si , X. Chen , W. Dai , X. Fu , X. Wang , Sens. Actuators, B 2012, 174, 449.

[38] E. Comini , G. Faglia , G. Sberveglieri , Sens. Actuators, B 2001, 78, 73.

[39] E. Espid , F. Taghipour , Sens. Actuators, B 2017, 241, 828.

[40] B. Fabbri , A. Gaiardo , A. Giberti , V. Guidi , C. Malagu , A. Martucci , M. Sturaro , G. Zonta , S. Gherardi , P. Bernardoni , Sens. Actuators, B 2016, 222, 1251.

[41] S.‐W. Fan , A. K. Srivastava , V. P. Dravid , Sens. Actuators, B 2010, 144, 159.

[42] H.‐Y. Li , J.‐W. Yoon , C.‐S. Lee , K. Lim , J.‐W. Yoon , J.‐H. Lee , Sens. Actuators, B 2018, 255, 2963.

[43] S. Park , S. An , H. Ko , S. Lee , C. Lee , Sens. Actuators, B 2013, 188, 1270.

[44] S. Park , S. An , Y. Mun , C. Lee , ACS Appl. Mater. Interfaces 2013, 5, 4285. 23627276 10.1021/am400500a

[45] J. D. Prades , R. Jimenez‐Diaz , F. Hernandez‐Ramirez , S. Barth , A. Cirera , A. Romano‐Rodriguez , S. Mathur , J. R. Morante , Sens. Actuators, B 2009, 140, 337.

[46] T. Wagner , C.‐D. Kohl , C. Malagu , N. Donato , M. Latino , G. Neri , M. Tiemann , Sens. Actuators, B 2013, 187, 488.

[47] J. Park , S. Yoon , K. Kang , S. Jeon , Small 2010, 6, 1981. 20715070 10.1002/smll.201000275

[48] F. Kayaci , S. Vempati , I. Donmez , N. Biyikli , T. Uyar , Nanoscale 2014, 6, 10224. 25056654 10.1039/c4nr01887g

