# Supplementary material for: Optically Activated 3D Thin‐Shell TiO2 for Super‐Sensitive Chemoresistive Responses: Toward Visible Light Activation
Source: Adv Sci (Weinh). 2020 Dec 3;8(3):2001883. doi: 10.1002/advs.202001883 (PMC7856904; doi:10.1002/advs.202001883)
Supplement: Supplementary file 1 — Supporting Information [file ADVS-8-2001883-s001.pdf]

© 2020 Wiley-VCH GmbH

ADVANCED SCIENCE
Open Access

Supporting Information

for *Adv. Sci.*, DOI: 10.1002/advs.202001883

Optically Activated 3D Thin-Shell TiO_2 for Super-sensitive Chemoresistive Responses: Toward Visible Light Activation

Donghwi Cho, Jun Min Suh, Sang-Hyeon Nam, Seo Yun Park, Minsu Park, Tae Hyung Lee, Kyoung Soon Choi, Jinho Lee, Changui Ahn, Ho Won Jang, Young-Seok Shim*, and Seokwoo Jeon**

Supporting Information

Optically Activated 3D Thin-Shell TiO_2 for Super-sensitive Chemoresistive Responses: Toward Visible Light Activation

Donghwi Cho†, Jun Min Suh†, Sang-Hyeon Nam, Seo Yun Park, Minsu Park, Tae Hyung Lee, Kyoung Soon Choi, Jinho Lee, Changui Ahn, Ho Won Jang, Young-Seok Shim*, and Seokwoo Jeon**

Experimental Section

Fabrication of a 3D polymeric template on an IDEs substrate: Interdigitated electrodes (IDEs) with 5 μm spacing were fabricated by depositing platinum (100 nm) and titanium (30 nm) on a SiO_2/Si substrate using photolithography and e-beam evaporation techniques.[3, 4] After cleaning the IDEs substrate by dipping and sonicating in acetone and ethanol in sequence, the substrate was dried and treated by air plasma (CUTEMP, Femtoscience) at a flow rate of 45 s.c.c.m., pressure of 40 mTorr, and power of 60 W for 120 s. Afterward, the edges of the electrodes were selectively concealed with commercial adhesives (3M tape) to avoid contamination during the following photoresist (PR) spin-casting step. First, a thin layer (~2 μm) of the PR (SU-8 2, Microchem) was spin-coated onto a plasma-treated substrate as an adhesion-promoting layer. After removing the pre-deposited tape, the adhesion layer was patterned into the open-window structure at the electrode pattern and hard-baked on a hot plate at 210 °C for 5 min. Then, a relatively thick layer (~10 μm) of the PR (SU-8 10, Microchem) was spin-coated on the adhesion layer again. The PR layer was carefully soft baked in 2 steps: 65 °C for 30 min and 95 °C for 30 min. Then, the substrate was induced to soft contact with a conformal phase mask designed to have a square array of holes (with a diameter of ~480 nm, a depth of ~400 nm, and a periodicity of ~600 nm).[17, 20, 24, 25] After the 3D nanopatterning process using the proximity-field nanopatterning (PnP) technique,

conventional lithographic procedures including a post-baking step with a 65 °C hot plate for 7 min, a developing step (SU-8 developer, Microchem), and a rinsing step with deionized water and a drying process were performed.

Material conversion into a 3D TiO_2: A thin TiO_2 layer (0–100 nm) was conformally deposited on the pre-fabricated 3D template by atomic layer deposition (ALD) technique at 90 °C (Atomic-Classic, CN1 Co., Ltd).[24] Tetrakis-dimethyl-amido titanium (TDMAT) (UP Chemical) was used as a precursor and H_2O was used as a reactant, respectively. The deposition cycles were controlled to modify the deposited TiO_2 thickness with the deposition rate of approximately 0.75 Å per cycle. Then, the 3D polymeric template was placed in a furnace at 500 °C to obtain the 3D TiO_2 hollow nanostructure by thermally decomposing the 3D polymeric template and annealing the TiO_2 in a single thermal step.

Characterization: The morphologies of the 3D TiO_2 samples were characterized using field-emission scanning electron microscopy (FESEM, S-4800, Hitachi) operating at an accelerating voltage of 5–10 kV. The structural features of the fabricated 3D TiO_2 were obtained by field emission transmission electron microscopy (FE-TEM) using a Tecnai F20 (FEI Company) at an accelerating voltage of 200 kV. The crystallographic analysis of the samples was performed by high-resolution powder X-ray diffraction (XRD) using SmartLab (RIGAKU). Elemental mapping measurements were collected via energy dispersive spectroscopy (EDS, Magellan 400, FEI). The absorbance measurements were performed using UV-VIS spectrophotometry (SolidSpec-3700, Shimadzu). The spectra of photoluminescence (PL) were collected by a fluoro-spectrophotometer using an F-7000 (Hitachi) with an excitation source for Xe lamp (150 W). The chemical bonding and core-electron binding energy of the 3D nanostructured TiO_2 film samples were investigated by in-situ X-ray photoelectron spectroscopy (XPS) using an Axis-Supra (Kratos) with automated

monochromatic X-ray source. To compensate for the surface charging, we used a low energy electron flood gun (charge neutralizer).

Gas sensing measurement: The gas-sensing measurement was conducted in a quartz tube with a gas injection line and a ventilation line. The breadboard with commercial LEDs (PL-UVA521CMK (Photron Co., Ltd), 513SBC3Z, 513UGC, 513UYC3 (Britestone)) was placed on top of a quartz tube with the help of plastic support. The gas injection was conducted using an automated mass flow controller repeating a sequence of dry air for 3000 s and the target gas (balanced with dry air, Sinjin Gases) for 500 s. The gas flow was maintained at 1000 s.c.c.m. throughout the whole measurements. The gas responses toward the target gas were precisely measured at a DC bias voltage of 5.0 V using a source meter (Keithley 2400). Additional experimental details about the gas-sensing measurement can be found in our previous works.[31]

Simulations: The optical modeling and calculations were performed by commercial finite differential time-domain (FDTD, Lumerical Solutions Ltd.). The PnP process with a square array phase mask (period: 600 nm) was simulated in the set of a 5-nm cubic mesh and refractive indices of each component (PDMS mask: 1.4, SU-8 photoresist: 1.66) with a 355 nm plane wave source assuming periodic boundaries. The resulting 3D interference image in photoresist was modeled to the 3D SU-8 polymer template by the level-set method to possess a fill factor of 50 %.[18] To consider the TiO_2 thin-shells on the template by ALD deposition, the thin-shell structures were generated to conformally cover the SU-8 template with the measured refractive index by ellipsometry. For the optical responses of the TiO_2 optical mazes, the electrical intensity field distributions of each model were calculated to visualize and further analyzed to select intensities only in the TiO_2 thin-shell regions.

Supplementary Figures

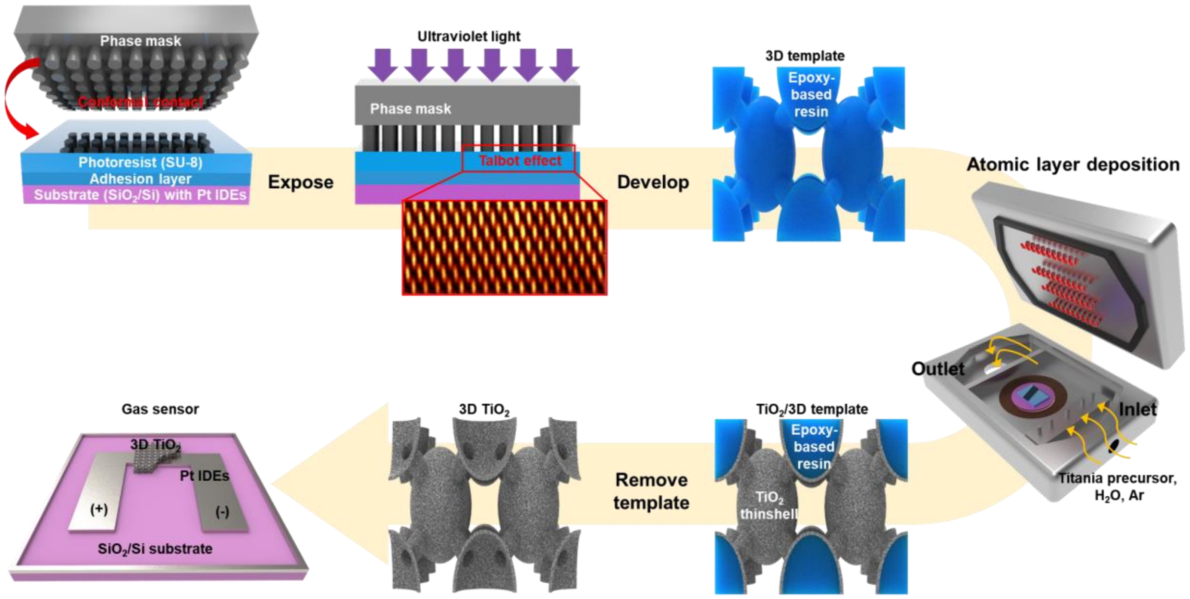

Figure S1. Schematic illustrations for the overall fabrication process of 3D TiO_2 gas sensor.

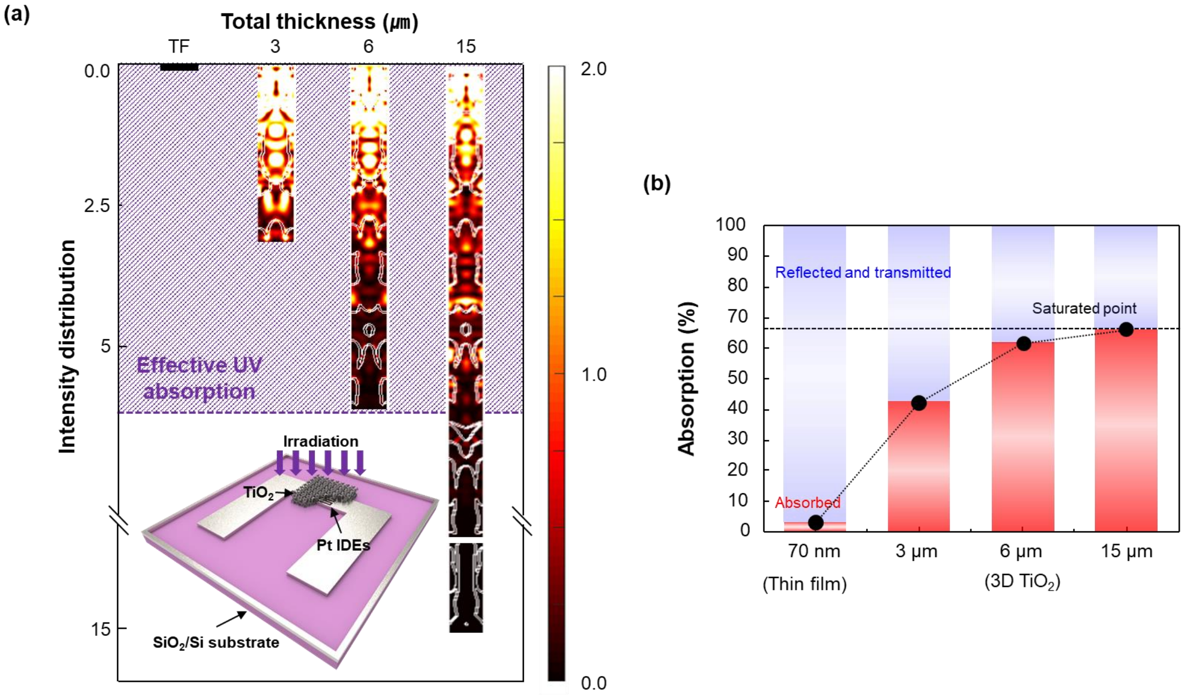

Figure S2. (a) Simulated depth profile of 3D TiO_2 with E-field distribution along the cross-section of the samples. (b) Calculated UV absorption depending on the total thickness of 3D TiO_2.

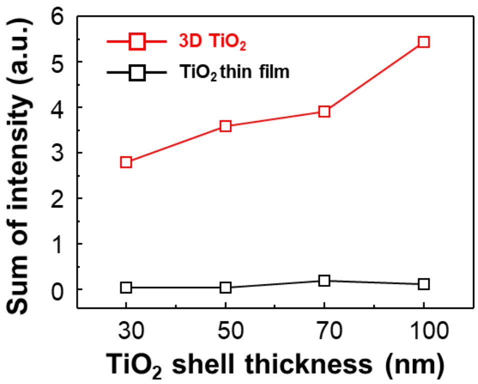

Figure S3. Simulated total sum of intensity for 3D TiO_2 and planar TiO_2 thin films.

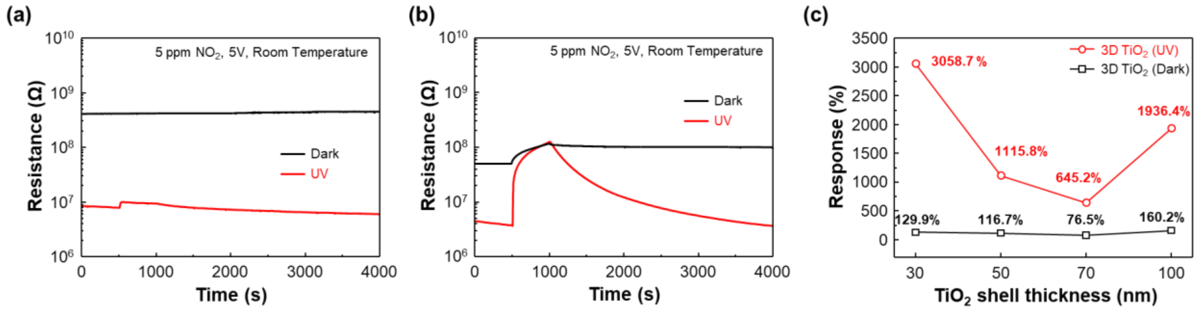

Figure S4. Resistance change plots of (a) planar TiO_2 thin film and (b) 3D TiO_2 with and without UV illumination. (c) Gas responses of 3D TiO_2 as a function of TiO_2 thin-shell thickness with and without UV illumination.

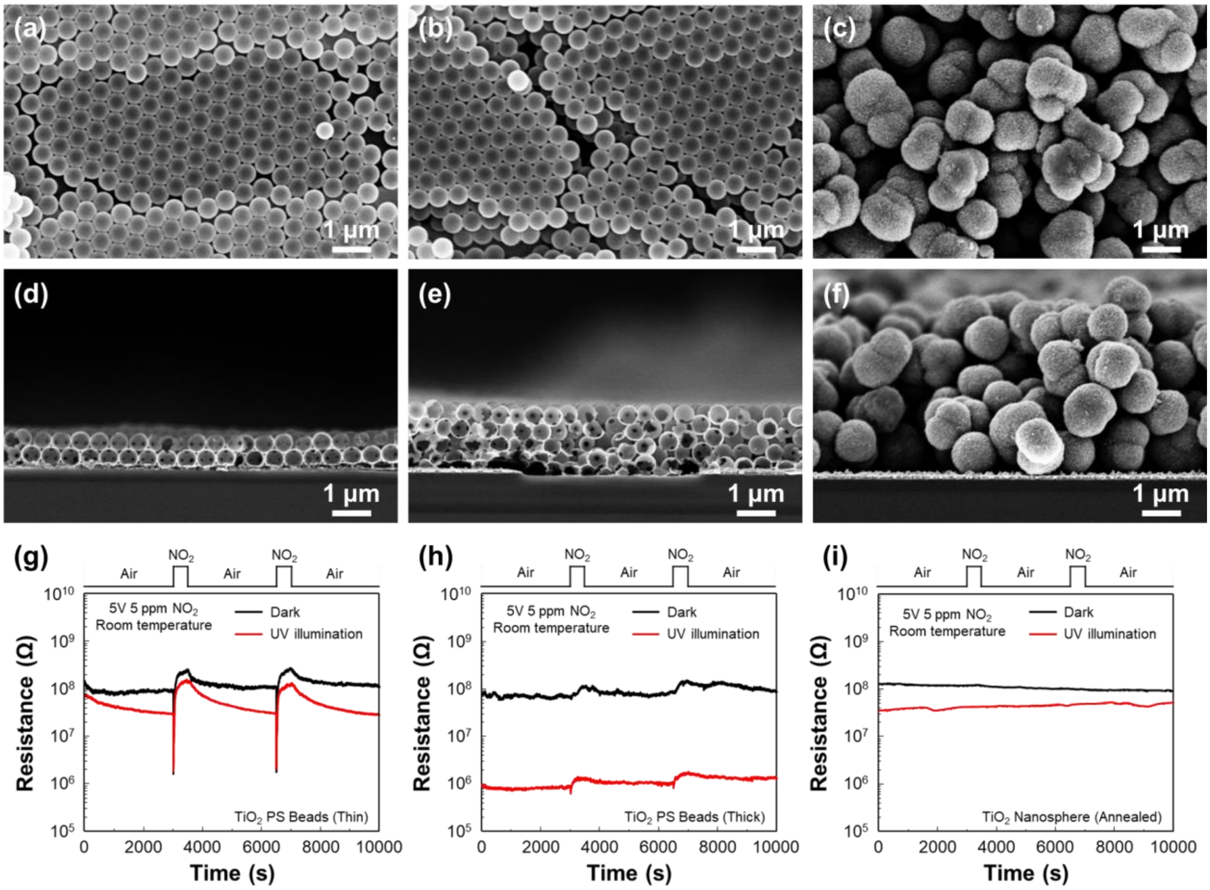

Figure S5. (a-c) Top-view and (d-f) cross-sectional view SEM images of (a, d) TiO_2 inverse opal with 1 μm thickness, (b, e) TiO_2 inverse opal with 2 μm thickness, and (c, f) self-assembled TiO_2 nanoparticles. (g-i) Gas sensing properties toward 5 ppm NO_2 under dark and UV-illuminated conditions of (g) TiO_2 inverse opal with small thickness, (h) TiO_2 inverse opal with large thickness, and (i) self-assembled TiO_2 nanoparticles.

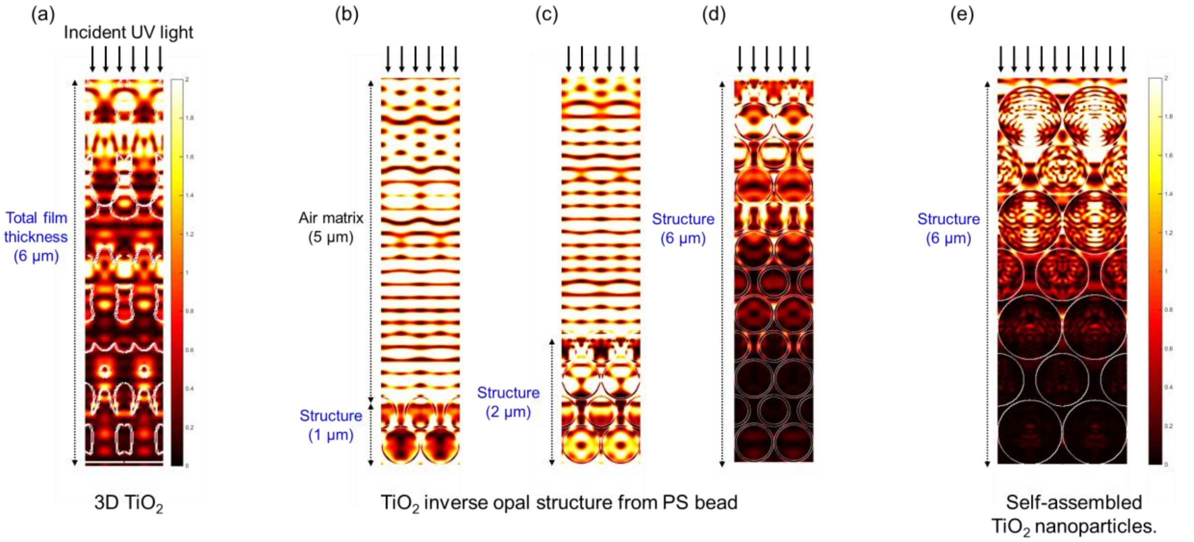

Figure S6. E-field intensity distribution for (a) 3D TiO_2, (b-d) TiO_2 inverse opal with (b) 1 μm thickness, (c) 2 μm thickness, and (d) 6 μm thickness, and (e) self-assembled TiO_2 nanoparticles simulated by finite element modeling. The TiO_2 shell thickness is fixed as 30 nm.

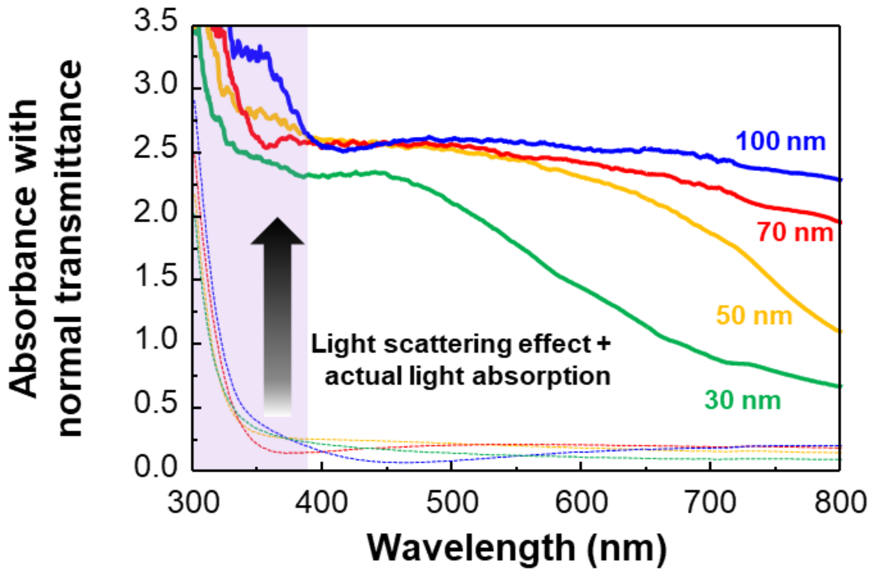

Figure S7. Absorbance results of 3D TiO_2 and planar TiO_2 thin film. The absorbance was calculated with measured normal transmittance values.

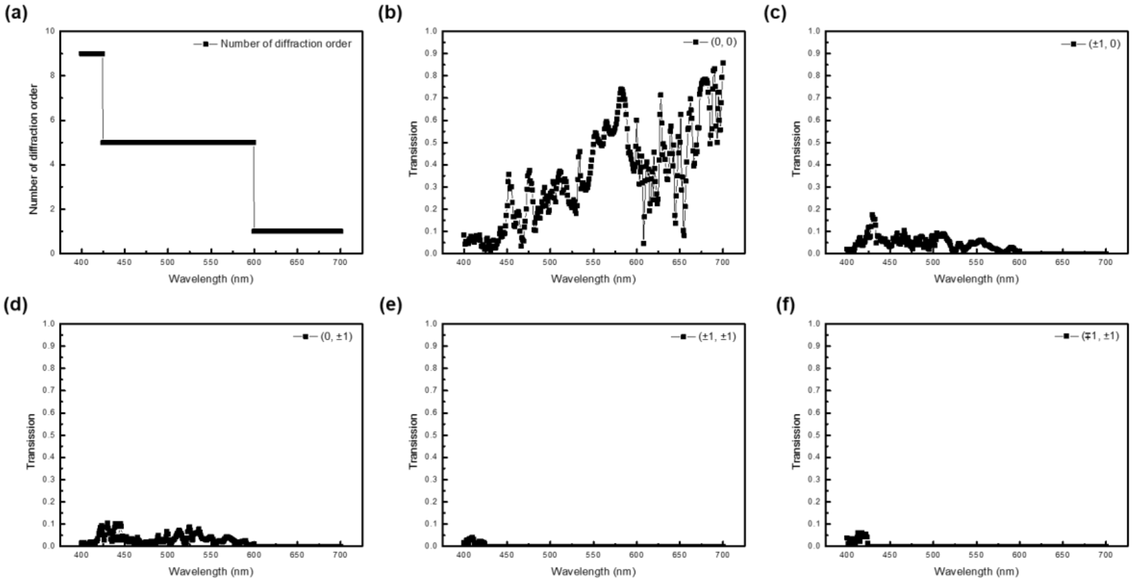

Figure S8. Calculated transmissions through 3D TiO_2. (a) Number of diffraction order through 3D TiO_2 and transmission at (b) 0th order, (c–d) 1st order, and (e–f) 2nd order of the diffracted lights.

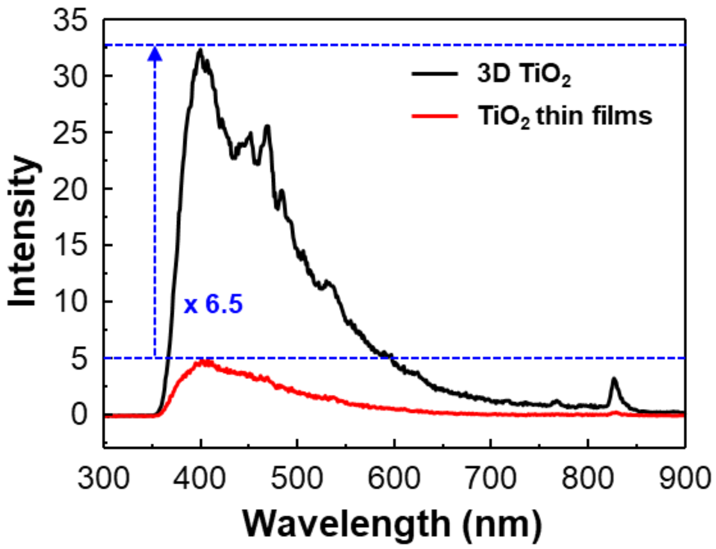

Figure S9. Comparison of PL spectra between 3D TiO_2 and planar TiO_2 thin film.

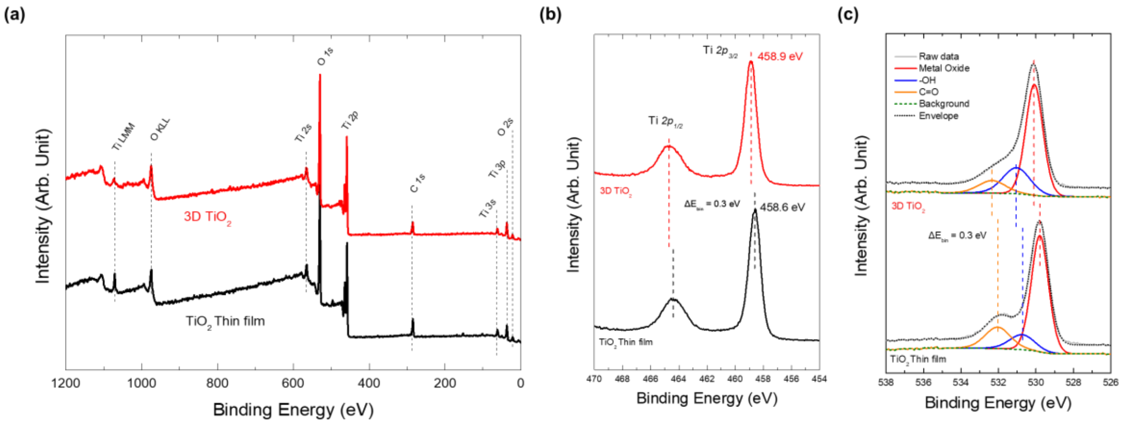

Figure S10. (a) Survey, (b) Ti 2*p* spectra, and (c) O 1*s* spectra from XPS analysis of the chemical bonding for 3D TiO_2 and the planar TiO_2 thin film.
